# Genomic sequencing of two isolates of *Ralstonia solanacearum* causing Sergipe facies and comparative analysis with Bugtok disease isolates

**DOI:** 10.1590/1678-4685-GMB-2020-0155

**Published:** 2020-11-06

**Authors:** Jéssica Rodrigues da Silva, Ana Karolina Leite Pais, Greecy Mirian Rodrigues Albuquerque, Adriano Márcio Freire Silva, Wilson José Silva, Valdir de Queiroz Balbino, Maria Esther Noronha Fonseca, Marco Aurélio Siqueira da Gama, Elineide Barbosa de Souza, Rosa de Lima Ramos Mariano

**Affiliations:** 1 Instituto Federal de Educação, Ciência e Tecnologia do Piauí - IFPI, Teresina, PI, Brazil.; 2 Universidade Federal Rural de Pernambuco - UFRPE, Departamento de Agronomia, Recife, PE, Brazil.; 3 Universidade Federal de Alagoas - UFAL, Departamento de Agronomia, Maceió, AL, Brazil.; 4 Universidade Federal de Pernambuco - UFPE, Departamento de Genética, Recife, PE, Brazil.; 5 Embrapa Hortaliças, Brasília, DF, Brazil.; 6 Universidade Federal Rural de Pernambuco - UFRPE, Departamento de Biologia, Recife, PE, Brazil.

**Keywords:** Banana tree, inflorescence infection, phylotype

## Abstract

*Ralstonia solanacearum* is the causal agent of Moko disease in bananas, which in the state of Sergipe in northeastern Brazil causes “Sergipe facies”. This disease induces atypical symptoms similar to those of Bugtok disease in the Philippines. This study was conducted to sequence, assemble, and annotate the genomes of the Sergipe facies-causing isolates SFC and IBSBF2570 (sequevar IIA-53) and compare their genomes with two representative isolates causing Bugtok disease. The genomes were sequenced and assembled, resulting in lengths of 5.58 Mb (SFC) and 5.46 Mb (IBSBF2570) in 185 and 174 contigs, respectively. The isolates of Sergipe facies and Bugtok disease showed similarities in their gene contents. We identified 5,668 information clusters, 3,752 of which were shared by all genomes (core genes). Moreover, 3,585 single-copy genes were identified. Isolates causing Bugtok disease exclusively shared 266 more information clusters than the isolates causing Sergipe facies. These results suggest that Sergipe facies and Bugtok disease isolates show high genomic similarity. However, the similarity is even greater between the Bugtok disease isolates. This may be because of their longer period of interaction compared to Sergipe facies isolates.

*Ralstonia solanacearum* is the causal agent of Moko disease in triploid bananas and *Heliconia* species (Hayward, 1994). Because of its heterogenicity, it is considered a species complex (Fegan and Prior, 2005) and has been classified into four phylotypes based on the geographical origins of the isolates and several sequevars, which are groups of strains with highly conserved regions in the endoglucanase gene (Fegan and Prior, 2006). Recently, three species were proposed in this species complex: *R. solanacearum*, *R. pseudosolanacearum*, and *R. syzygii* (Safni *et al.*, 2014). The isolates causing Moko in bananas were classified as *R. solanacearum* belonging to phylotype II and subdivided into IIA and IIB (Fegan and Prior, 2006), originating in the Americas (Prior and Fegan, 2005), and sequevars IIA-6, IIA-24, IIA-41, IIA-53, IIB-3, IIB-4, and IIB-25 (Fegan and Prior, 2005; 2006; Albuquerque *et al.*, 2014).

Typical symptoms of Moko include yellowing and withering of the leaves, caused by an infection that begins in the rhizomes and moves towards the pseudostem. The fruits become deformed, black, and stunted. Banana plants close to maturity may not display any obvious symptoms but the internal fruit pulp can still show dry rot and the plants may die. However, in the state of Sergipe in northeastern Brazil, there is a variant of Moko disease known as “Sergipe facies”. This disease is caused by endemic isolates of *R. solanacearum* within sequevar IIA-53, which initiate symptoms in the inflorescences and progress to dry rot and fruit deformation without wilting. These symptoms are similar to those caused by Bugtok disease, which occurs in the Philippines (Albuquerque *et al*., 2014). The latter mainly attacks the banana cultivars 'Saba' and 'Cardaba' and is associated with isolates from sequevar IIB-3 (Thwaites *et al*., 2000).

The objective of this study was to sequence, assemble, and annotate the genomes of two isolates of sequevar IIA-53 of *R. solanacearum*, the causal agent of Sergipe facies (SFC and IBSBF2570), compare them with the genomes of isolates of sequevar IIB-3, the causal agent of Bugtok disease (CIP417 and Molk2), and deposit these sequences into the United States National Center for Biotechnology Information (NCBI) database.

The SFC and IBSBF2570 isolates were grown in 2,3,5-triphenyl tetrazolium chloride medium (Kelman, 1953) at 28°C for 48 h, and then DNA was extracted using the PureLink^®^ Genomic DNA kit (Thermo Fisher Scientific, Waltham, MA, USA) following the manufacturer's instructions. The DNA was quantified using a spectrophotometer (BioDrop, Thermo Fisher Scientific) and subjected to electrophoresis on 0.8% agarose gels to assess its integrity.

Sequencing libraries were prepared using the Illumina Nextera DNA Flex Prep Kit (Illumina, San Diego, CA, USA). Sequencing was performed on a MiSeq-2500 Platform (Illumina) to generate pair-ended reads at the ESALQ-USP Functional Genomics Center. The quality of the reads was verified using FastQC (Andrews, 2010), sequence filtering was performed using FASTX-Clipper v. 0.0.13, and sequence trimming was performed using Sickle v. 1.33 (Joshi and Fass, 2011).

*De novo* assembly was performed using the Unicycler pipeline (Wick *et al*., 2017) and ABACAS software v.1.3.1 (Assefa *et al*., 2009) for alignment with the PROmer and NUCmer algorithms. The complete genomes of the UW163 (GCF_001587135.1) and Po82 (GCF_000215325.1) isolates (both sequevar IIB-4) of *R. solanacearum* were used as alignment references. Contig evaluation and selection of the scaffolds formed from alignments showing the lowest number of Ns and the largest number of predicted genes were performed using QUAST software v.5.0.2 (Gurevich *et al*., 2013). BUSCO software (Seppey *et al*., 2019; implemented in QUAST) was used to identify single-copy orthologs and analyze the conservation of the gene contents. The alignment between the Sergipe facies (SFC and IBSBF2570) and Bugtok (CIP417 and Molk2) causing isolates genomes was visualized using the Circos package (Krzywinski *et al*., 2009) in QUAST. Gene predictions and automatic annotation of proteins present in the assembled scaffolds were performed using the RAST platform (Aziz *et al*., 2008), followed by analysis of orthologous gene clusters using OrthoVenn (Xu *et al*., 2019).

After treatment of the raw reads, the genomes were assembled at the contig level to generate sequences of 5.5 Mb (SFC) and 5.4 Mb (IBSBF2570) in 185 and 174 contigs, respectively (Table 1). The assembly showed little variation in N50 (i.e., the shortest contig length required for 50% genome coverage) between the genomes and a high rate of conservation in the gene content (98%), indicating a reliable assembly. The lengths of the sequences and their GC content (66%) were consistent with the genome sequences of the Moko-causing isolates deposited in the NCBI database. Scaffold construction was based on the PROmer algorithm using the *R. solanacearum* isolates Po82 and UW163 as references for the chromosome and megaplasmid, respectively.

**Table 1 -:** Genome characteristics of isolates SFC and IBSBF2570 of *Ralstonia solanacearum* causing Sergipe facies disease.

Features	SFC		IBSBF2570	
Coverage	151.35		156.75	
Genomes size (bp)^a^	5.578.372		5.455.860	
G + C content (%)	66.61		66.59	
Total contig number	185		174	
N50	94.771		94.853	
BUSCO (%)	98.65		98.65	
Coding sequence	4,937		4,862	
Subsytems number	351		345	
RNAs	52		51	
Genome size (bp)^b^	5.722.671		5.713.471	
	Chrom^c^	Plasmid^d^	Chrom	Plasmid
Reference isolates	^e^Po82	^e^UW163	Po82	UW163
Genome size (bp)^b^	3.630.670	2.092.001	3.656.700	2.056.771

a Genome size before alignment;

b Genome size after alignment;

c Chromosome;

d Megaplasmid;

e Po82 e UW163 (sequevar IIB-4) - Isolates of *R. solanacearum* used as a reference in the alignment of genomic sequences.

Based on the annotation of the isolates causing Sergipe facies, proteins were distributed into functional groups, and approximately 65% of the encoded proteins concentrated into seven subsystems: I- amino acids and derivatives (17.8%); II- carbohydrates (10.1%); III- cofactors, vitamins, protein groups, and pigments (10.0%); IV- protein metabolism (8.4%); V- membrane transport (8.3%); VI- fatty acids, lipids, and isopropenoids (5.5%); and VII- breathing (5.2%). Isolates causing Bugtok disease showed similar results, with a slightly different order of subsystem representativeness: I- amino acids and derivatives (16.7%); II- carbohydrates (10.3%); III- membrane transport (9.6%); IV- cofactors, vitamins, protein groups, and pigments (9.6%); V- protein metabolism (9.5%); VI- breathing (5.0%); and VII- fatty acids, lipids, and isopropenoids (4.2%; Figure 1). Because of the similarities between the atypical symptoms of the Sergipe facies and Bugtok diseases, which initiate from inflorescences in both cases, a high degree of genome similarity was expected. 

**Figure 1 -:**
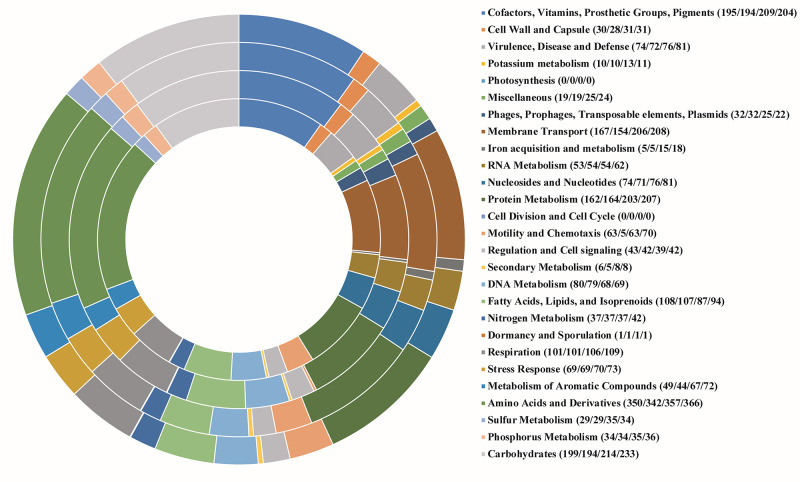
Distribution of the subsystem category and gene coverage of isolates of *Ralstonia solanacearum* causing Sergipe facies (SFC and IBSBF2570) and Bugtok disease (CIP417 and Molk2). The graphic representation shows the grouping of genes from the SFC, IBSBF2570, CIP417, and Molk2 genomes from the inside to out. The numbers between parentheses in the legend represent the number of genes in each subsystem in the same order.

Visualization of the alignment built based on the genomic sequences of the two reference isolates (Po82 and UW163) revealed the absence of GC content in two regions of Po82 (Figure 2A, outermost circle) and four regions of UW163 (Figure 2B, outermost circle) in all isolates. The assembly strip individually highlighted the incompatibility (taller columns = more incompatibility) and gene density (higher green or red intensity = higher gene density) of all isolates, enabling identification of low-density regions in the isolates causing Sergipe facies (SFC and IBSBF2570). This was not observed in isolates causing Bugtok disease, nor was it correlated with gene density (Figure 2).

**Figure 2 -:**
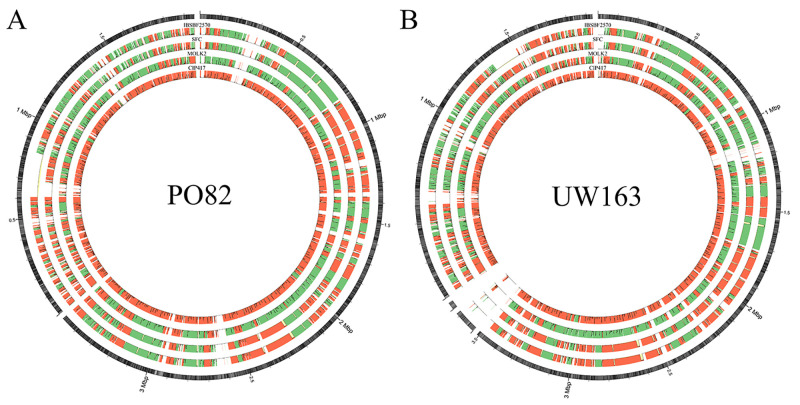
Visualization of the alignment of *Ralstonia solanacearum* isolates causing Sergipe facies (SFC and IBSBF2570) and Bugtok disease (CIP417 and Molk2) with the reference isolates Po82 (A) and UW163 (B) of *R. solanacearum*. The outer circle represents the reference sequence with GC (%) heatmap [from 0% (white) to 77% (black)]. Assembly tracks are combined with the display of incompatibilities: taller columns indicate a higher rate of incompatibility. Darker colors indicate higher gene density. The green color represents similar contigs to the reference and red color indicates misassembled contigs.

In a Venn diagram of the isolates causing Sergipe facies and Bugtok disease, 5,668 information clusters were identified, of which 3,752 were shared between all genomes (core genes), and 3,585 were single copy genes (Figure 3; Table S1 ). The isolates that cause Bugtok disease symptoms exclusively shared 970 (17.1%) clusters involved in 76 biological processes and 17 molecular functions and were associated with seven cellular components. The isolates that cause Sergipe facies shared 704 (12.4%) clusters involved in 65 biological processes and 11 molecular functions and were associated with five cellular components (Table S1). The results indicate that the isolates causing Bugtok disease showed greater genetic similarity, as they exclusively shared 266 more information clusters than those causing Sergipe facies. The greater genetic similarity between Bugtok isolates may be related to the increased interaction time between the isolates, considering that Bugtok disease was first reported in 1965 (Soguilon *et al*., 1995, Thwaites *et al*., 2000), whereas Sergipe facies was first reported in 2014 (Albuquerque *et al*., 2014). However, other factors cannot be ruled out, particularly with regard to living organisms interacting freely in nature.

**Figure 3 -:**
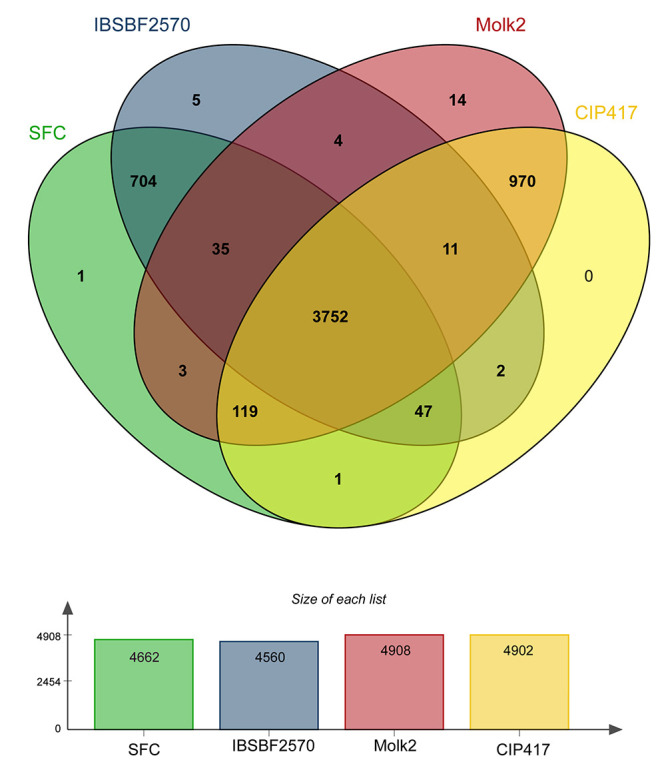
Venn diagram showing clusters present in the genome of isolates of *Ralstonia solanacearum* causing Sergipe facies (SFC and IBSBF2570) and Bugtok disease (CIP417 and Molk2).

The sequencing, assembly, and annotation of the genomes of isolates of *R. solanacearum* causing Sergipe facies (sequevar IIA-53) provide a foundation for further research aiming to understand the interactions between *R. solanacearum* isolates and banana. SFC and IBSBF2570 are the only northeast Brazilian isolates and first isolates causing Sergipe facies whose genomes have been sequenced.

The genomes have been deposited in DDBJ/EMBL/GenBank under accession numbers CP026090 and CP026091 (for the chromosome and megaplasmid of isolate IBSBF2570, respectively) and CP026092 and CP026093 (for the chromosome and megaplasmid of isolate SFC, respectively).

## Supplementary material

The following online material is available for this article:

Table S1 - Annotation of orthologous genes present in the genomes of *Ralstonia solanacearum* isolates causing Sergipe facies (SFC and IBSBF2570) and Bugtok disease (CIP417 and Molk2).
