# Supplementary material for: Genomic sequencing of two isolates of Ralstonia solanacearum causing Sergipe facies and comparative analysis with Bugtok disease isolates
Source: Genet Mol Biol. 2020 Nov 6;43(4):e20200155. doi: 10.1590/1678-4685-GMB-2020-0155 (PMC7783953; doi:10.1590/1678-4685-GMB-2020-0155)
Supplement: Table S1 - Annotation of orthologous genes present in the genomes of Ralstonia solanacearum isolates causing Sergipe facies (SFC and IBSBF2570) and Bugtok disease (CIP417 and Molk2). [file 1415-4757-GMB-43-4-e20200155-s1.pdf]

Genetics and Molecular Biology
DOI: https://doi.org/10.1590/1678-4685-GMB-2020-0155

Suplementary material to "Genomic sequencing of two isolates of *Ralstonia solanacearum* causing Sergipe facies and comparative analysis with Bugtok disease isolates"

Table S1 – Annotation of orthological genes present in the genomes of *Ralstonia solanacearum* isolates from the causal agents of Sergipe Facies (SFC and IBSBF2570) and Bugtok disease (CIP417 and Molk2).

	Genomes of Bugtok and Sergipe facies	
Orthologues clusters	5,668	
Core genes	3,752	
Single copy	3,585	
Exclusive clusters	Bugtok	Sergipe facies
Total	970	704
Biological processes	76	65
Molecular functions	17	11
Cellular components	7	9
